# Young Age Is Associated with Improved Survival After Resection of Non-Metastatic Pancreatic Ductal Adenocarcinoma

**DOI:** 10.3390/life16091499

**Published:** 2026-09-08

**Authors:** Ingrid Garajová, Bing Wang, Inna Markovna Chen, Carsten Palnæs Hansen, Annalisa Comandatore, Nina Fokter Dovnik, Radim Nemecek, Michal Eid, Marko Hojnik, Damjan Sisinger, Tomaž Rojko, Fabio Gelsomino, Stefania De Lorenzo, Geert Kazemier, Luca Morelli, Elisa Giovannetti

**Affiliations:** 1 Medical Oncology Unit, University Hospital of Parma, 43125 Parma, Italy; 2 Laboratory Medical Oncology, Department of Medical Oncology, Cancer Center Amsterdam, Amsterdam University Medical Center, Vrije Universiteit Amsterdam, 1081 HV Amsterdam, The Netherlands; b.wang1@amsterdamumc.nl (B.W.);; 3 Department of Oncology, Copenhagen University Hospital–Herlev and Gentofte Hospital, 2730 Copenhagen, Denmark; inna.chen@regionh.dk; 4 Department of Surgery and Transplantation, Rigshospitalet, Copenhagen University Hospital, 2100 Copenhagen, Denmark; carsten.palnaes.hansen@regionh.dk; 5 General Surgery Unit, Department of Translational Research and New Technologies in Medicine and Surgery, University of Pisa, 56126 Pisa, Italy; 6 Department of Medical Oncology, University Medical Centre Maribor, 2000 Maribor, Slovenia; 7 Department of Comprehensive Cancer Care, Masaryk Memorial Cancer Institute and Faculty of Medicine, Masaryk University, 65653 Brno, Czech Republic; 8 Department of Internal Medicine, Hematology and Oncology, University Hospital Brno, Faculty of Medicine, Masaryk University, 62500 Brno, Czech Republic; eid.michal@fnbrno.cz; 9 Department of Pathology, University Medical Centre Maribor, 2000 Maribor, Slovenia; 10 Department of Oncology and Hematology, University Hospital of Modena, 41124 Modena, Italy; 11 Oncology Unit, Azienda USL Bologna, 40124 Bologna, Italy; 12 Department of Surgery, Amsterdam UMC, University of Amsterdam, 1105 AZ Amsterdam, The Netherlands; 13 Cancer Center Amsterdam, 1105 AZ Amsterdam, The Netherlands; 14 Cancer Pharmacology Laboratory, AIRC Start-Up Unit, Fondazione Pisana per la Scienza, 56126 Pisa, Italy

**Keywords:** pancreatic ductal adenocarcinoma, early-onset pancreatic cancer, age, CA19-9, surgery, overall survival, prognosis

## Abstract

In pancreatic ductal adenocarcinoma (PDAC), clinicians often ask whether young age is associated with more aggressive disease or patients more likely to tolerate curative-intent multimodality treatment. The prognostic meaning of age after surgery remains uncertain. We analyzed a retrospective multicenter surgical cohort of patients with resected non-metastatic PDAC. Candidate clinical variables were evaluated in univariable Cox models. The prespecified primary multivariable model included age, sex, N status, T stage, and CA19-9 status. Adjuvant therapy was evaluated in a sensitivity model. The cohort included 696 surgical patients; 70 (10.1%) were aged < 55 years. Median overall survival was 34.4 months in patients aged < 55 years versus 22.0 months in those aged ≥ 55 years (log-rank *p* = 0.019). In the primary multivariable model, age < 55 years remained independently favorable (HR 0.66, 95% CI 0.49–0.89; *p* = 0.007). Elevated CA19-9 value independently predicted worse survival (HR 1.39, 95% CI 1.13–1.71; *p* = 0.002). The sensitivity model confirmed favorable associations for young age and not elevated CA19-9 value. In surgically managed PDAC patients, young age and normal CA19-9 value carried independent favorable prognostic information. Prognosis was also shaped by nodal status, T stage, and receipt of adjuvant therapy.

## 1. Introduction

Pancreatic ductal adenocarcinoma (PDAC) is usually diagnosed in older adults, yet a clinically meaningful minority of patients present at a younger age. For the treating clinician, younger age creates a practical tension: it may raise concern for distinct tumor biology and hereditary predisposition, but it may also identify patients with better physiological reserve, greater access to intensive treatment, and a higher likelihood of completing multimodality therapy. This tension is increasingly relevant because population-level analyses suggest that early-onset pancreatic cancer is rising in several countries and registries [1,2,3]. These epidemiologic signals have intensified interest in early-onset PDAC as a separate clinical entity. However, incidence trends alone do not establish whether younger age independently worsens prognosis after curative-intent surgery.

Prior clinical studies have produced heterogeneous results. Some analyses indicate that younger patients may present with more advanced or biologically distinctive disease, but they may also receive more aggressive treatment and achieve survival outcomes that are similar or superior to those of older patients [4,5,6,7]. In the resected setting, Leonhardt et al. showed that outcomes in early-onset pancreatic cancer were primarily driven by stage and margin status [8]. Mughal et al. further examined resected early-onset PDAC through clinical and molecular data, supporting the idea that young-onset disease is a relevant subgroup but not one reducible to chronological age alone [9]. Some authors also argue against interpreting chronological age as a purely oncologic variable. In the German StuDoQ|Pancreas registry, age was strongly associated with major complications, postoperative mortality, and failure-to-rescue after pancreatic surgery [10]. Conversely, survival analyses across age strata confirm that older age groups often have poorer survival, but these studies may reflect perioperative vulnerability, treatment delivery, and competing risks in addition to cancer biology [11].

Biomarker adjustment is particularly important in this setting. CA19-9 is embedded in PDAC practice as a prognostic and monitoring marker, and elevated CA19-9 value has repeatedly been associated with inferior survival after resection [12,13,14,15,16]. Because CA19-9 may reflect tumor burden and occult systemic disease, failure to account for it may overattribute prognostic differences to age. We therefore used the conventional clinical upper limit of normal, 37 U/mL, to classify CA19-9 as normal versus elevated.

We analyzed a multicenter cohort of surgically managed non-metastatic PDAC to test whether age < 55 years was independently associated with overall survival. Candidate variables were restricted to clinically available factors: age, sex, T stage, N status, CA19-9 status, and receipt of adjuvant therapy. The primary model focused on baseline clinical and pathological prognostic factors and CA19-9. Adjuvant therapy was analyzed separately as a sensitivity variable because it is delivered after surgery and is influenced by postoperative recovery, early progression, and treatment selection.

## 2. Materials and Methods

Study design and cohort. This was a retrospective multicenter surgical cohort study. Patients with documented M1 disease as well as missing follow-up data were excluded. The pooled cohort comprised consecutive patients undergoing curative-intent resection for histologically confirmed PDAC at several European participating centers (Italy, Slovenia, Czech Republic, Denmark, The Netherlands). Clinical and pathological variables were harmonized before analysis using predefined common definitions. Patients were included between January 2012 and May 2023 across multiple European centers, with a median follow-up of 56 months. Differences in clinical practice across participating centers may have existed; however, clinical and pathological variables were harmonized using predefined common definitions to improve comparability across the study population. The study was conducted in accordance with the Declaration of Helsinki and was approved by the Ethics Committees of all participating centers.

Exposure, biomarker, treatment, and endpoint. The primary exposure was age categorized as <55 versus ≥55 years. This cutoff was selected a priori to define a clinically younger-onset surgical subgroup while also reflecting translational evidence suggesting age-related molecular differences around 55 years; the conventional <50-year cutoff used in many database studies was retained as a sensitivity analysis. CA19-9 was classified as low/normal (≤37 U/mL) or elevated (>37 U/mL) using the standard clinical upper limit of normal. Adjuvant therapy was recorded as received or not received. Overall survival, defined as time from surgery to death or last follow-up, was the primary endpoint.

Statistical analysis. Kaplan–Meier curves and log-rank tests were used to compare overall survival between groups. Each candidate variable was first evaluated in a univariable Cox proportional hazards model. The prespecified primary multivariable Cox model included age group, sex, N-positive disease, T stage, and CA19-9 group. Adjuvant therapy was added only in a prespecified sensitivity model because it is a postoperative treatment variable. A further sensitivity analysis was prespecified using a <50-year age threshold, consistent with the most widely adopted definition of early-onset PDAC in database studies and meta-analyses to assess the robustness of the primary age association across cutoff definitions (4, 5, 7). Hazard ratios (HRs) are reported with 95% confidence intervals (CIs) and two-sided *p*-values. Complete-case analysis was used for multivariable models; 22 of 696 patients (3.2%) were excluded due to missing covariate data. Given this low proportion and absence of a systematic missingness pattern, complete-case analysis was considered appropriate and formal multiple imputation was not applied. The proportional hazards assumption was verified for each model term using scaled Schoenfeld residuals. Analyses were performed using R (version 4.3.3; R Foundation for Statistical Computing, Vienna, Austria).

## 3. Results

### 3.1. Patient Characteristics

The study cohort included 696 patients who underwent surgery for PDAC; 70 patients (10.1%) were aged < 55 years and 626 (89.9%) were aged ≥ 55 years. Patients aged < 55 years were less frequently female than those aged ≥ 55 years (35.7% vs. 50.2%; *p* = 0.022). A total of 524 deaths were recorded during follow-up. Median follow-up was 56.0 months. CA19-9 was available in 639 patients (91.8%). Baseline CA19-9 values were similar between age groups: median 118.7 U/mL (IQR 32.2–373.0) in patients aged < 55 years and 157.0 U/mL (IQR 28.4–681.0) in patients aged ≥ 55 years (*p* = 0.411). The proportion with elevated CA19-9 (>37 U/mL) was similar between groups (67.6% vs. 70.9%; *p* = 0.575). Patient characteristics are summarized in Table 1.

### 3.2. Survival Analysis by Age and CA19-9

**Figure 1:**
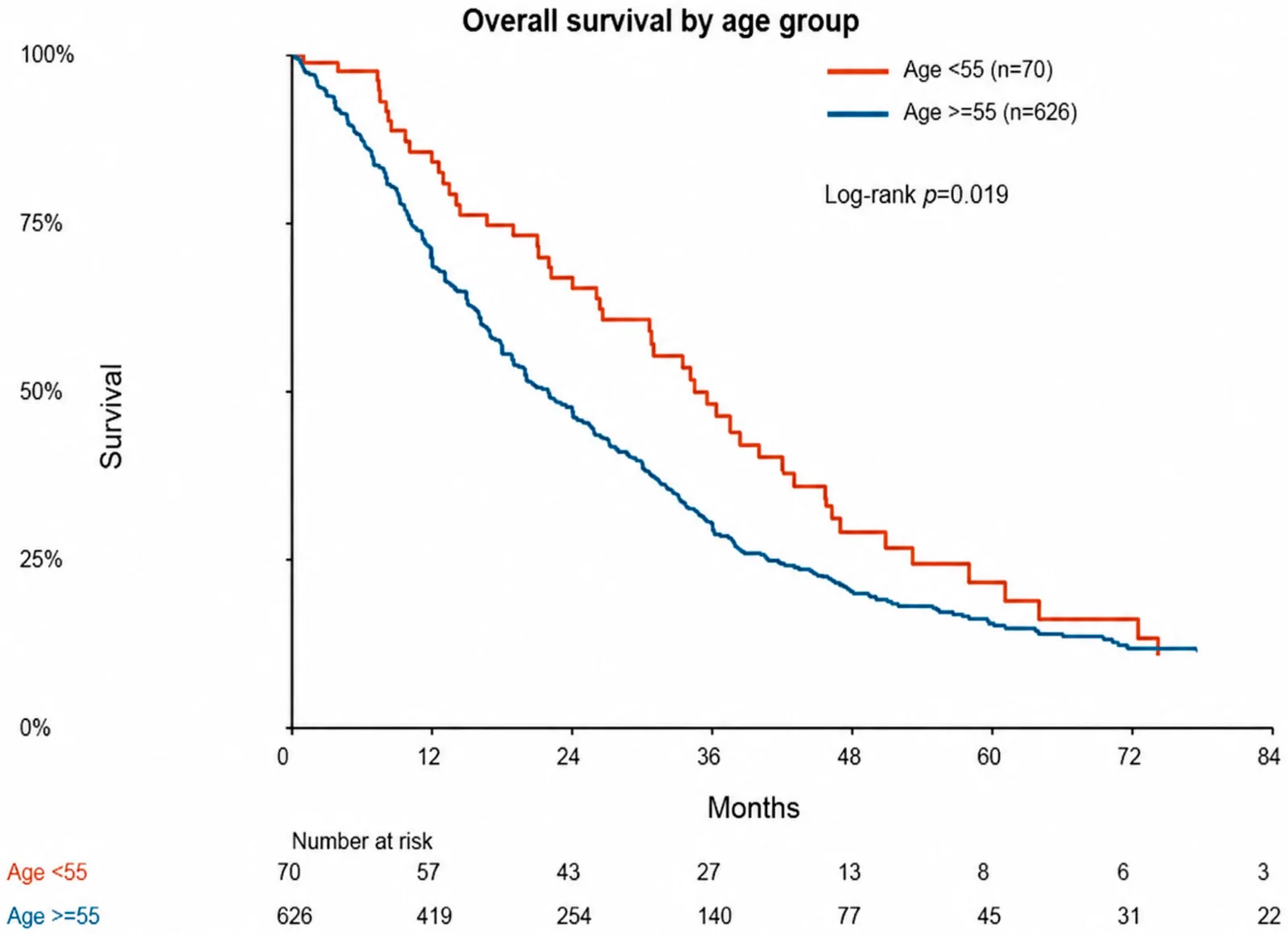
Kaplan–Meier overall survival curves by age group (<55 years vs. ≥55 years) in the surgical M0 cohort (*n* = 696; log-rank *p* = 0.019).

**Figure 2:**
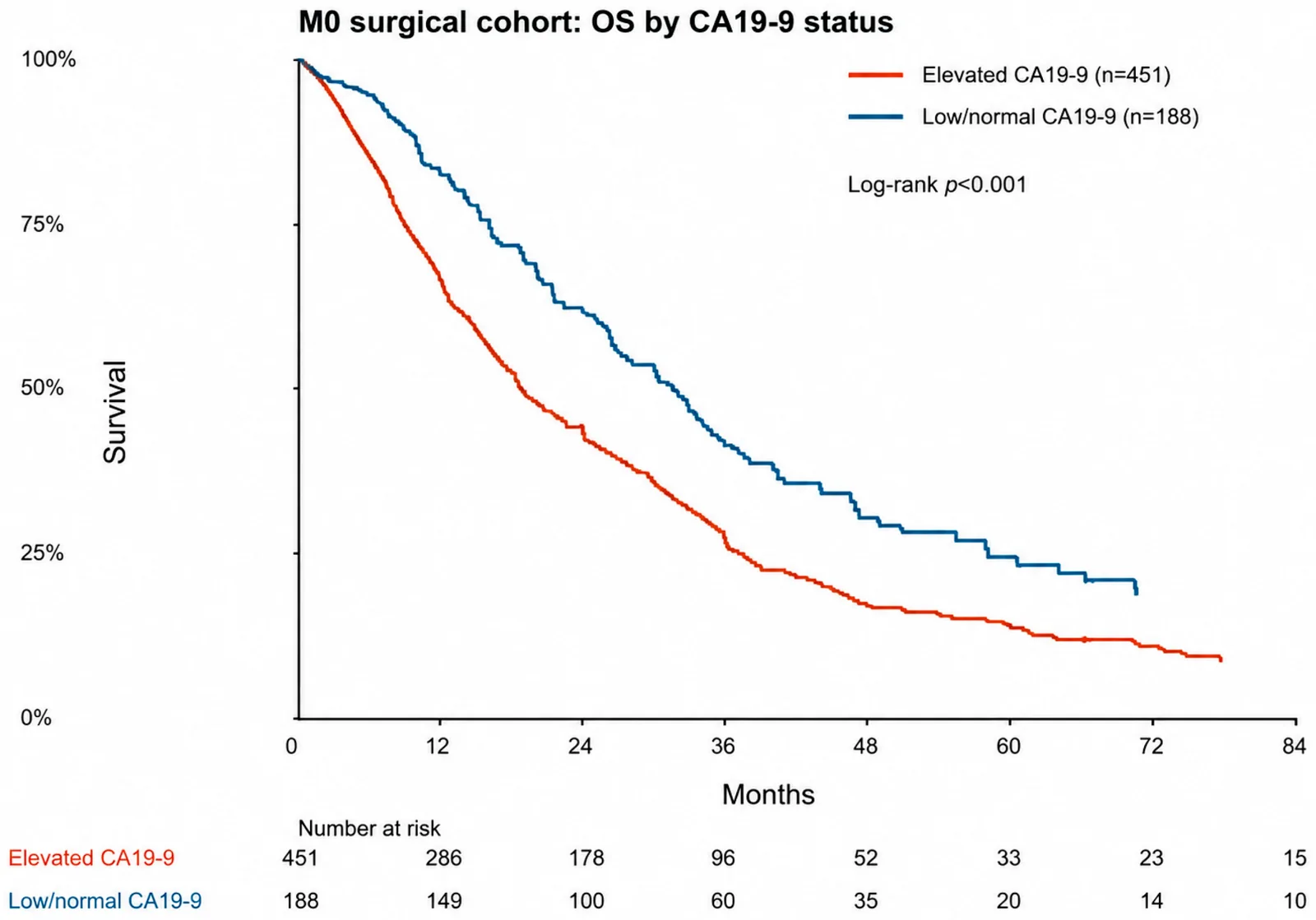
Kaplan–Meier overall survival curves by CA19-9 status (>37 U/mL vs. ≤37 U/mL; *n* = 639; log-rank *p* < 0.001). Tick marks indicate censored observations.

Kaplan–Meier analysis showed significantly longer overall survival in patients aged < 55 years compared with those aged ≥ 55 years (median 34.4 vs. 22.0 months; log-rank *p* = 0.019; Figure 1). Elevated CA19-9 (>37 U/mL) identified a subgroup with substantially worse survival: median OS 19.0 months for CA19-9 > 37 U/mL versus 31.5 months for CA19-9 ≤ 37 U/mL (log-rank *p* < 0.001; Figure 2). Survival by CA19-9 status is detailed in Table 2. In an exploratory analysis across four age categories (<55, 55–64, 65–74, and ≥75 years), median OS was 34.4, 25.3, 24.0, and 17.1 months, respectively (overall log-rank *p* < 0.001; Table S4). In the adjusted Cox model using < 55 years as the reference, the highest mortality risk was observed in patients ≥ 75 years (HR 2.12, 95% CI 1.52–2.96; *p* < 0.001), while the 55–64-year group showed a modestly higher risk (HR 1.41, 95% CI 1.01–1.99; *p* = 0.046) and the 65–74-year group was not statistically significant (HR 1.31, 95% CI 0.96–1.80; *p* = 0.092).

### 3.3. Univariable Cox Models

In univariable Cox regression, age < 55 years (HR 0.71, 95% CI 0.53–0.95; *p* = 0.022), N-positive disease (HR 1.59, 95% CI 1.29–1.97; *p* < 0.001), T stage (HR 1.27, 95% CI 1.17–1.39; *p* < 0.001), elevated CA19-9 (HR 1.55, 95% CI 1.27–1.90; *p* < 0.001), and adjuvant therapy (HR 0.53, 95% CI 0.43–0.65; *p* < 0.001) were each significantly associated with overall survival. Female sex was not significant (HR 0.87, 95% CI 0.73–1.04; *p* = 0.120). The full results are in Table 3.

### 3.4. Multivariable Cox Models

**Figure 3:**
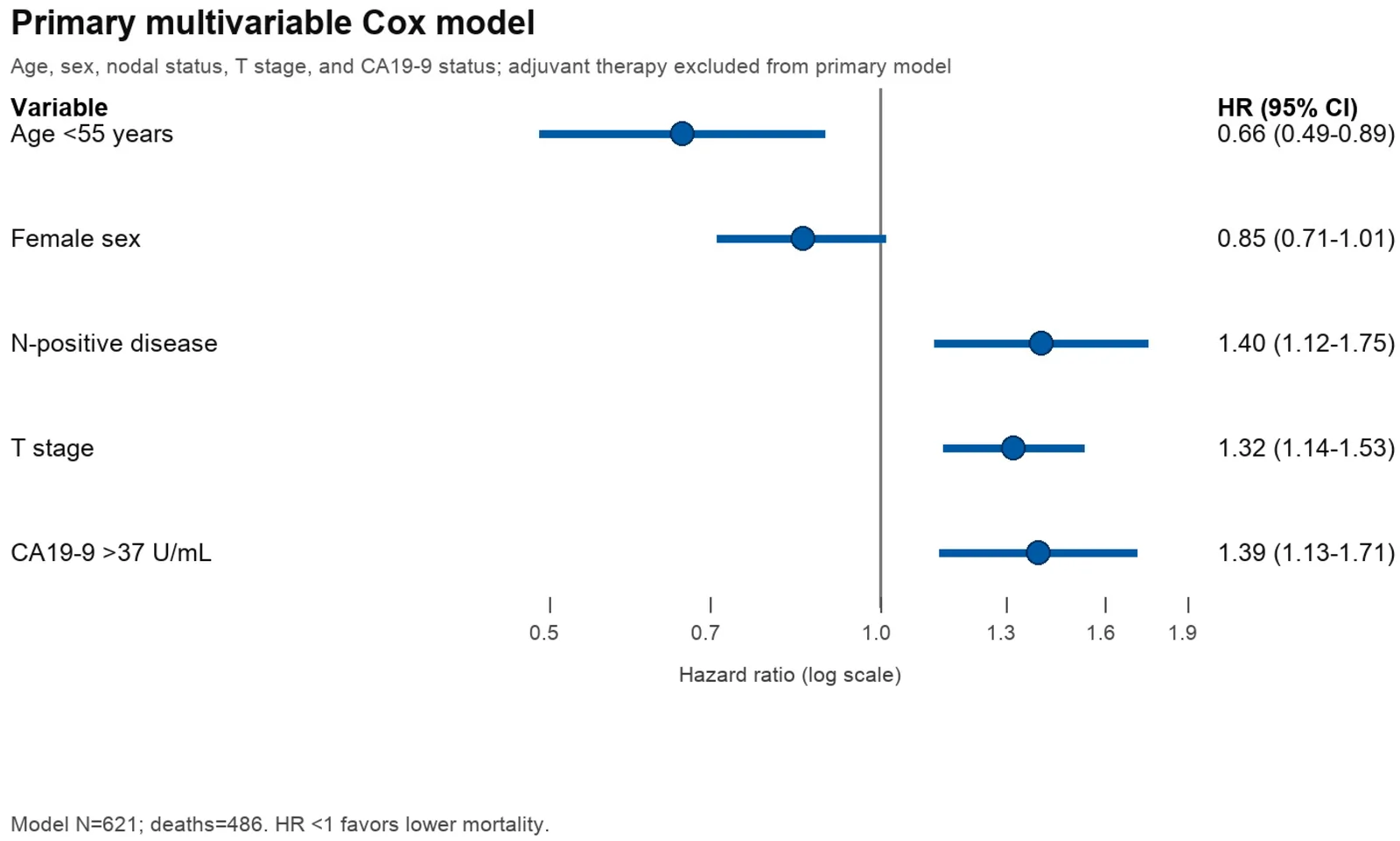
Forest plot of the primary multivariable Cox model (*N* = 621). Squares indicate hazard ratios; horizontal bars indicate 95% confidence intervals. HR < 1 indicates lower mortality risk; HR > 1 indicates higher mortality risk.

In the primary multivariable model (*N* = 621; deaths = 486), age < 55 years remained independently associated with lower mortality risk (HR 0.66, 95% CI 0.49–0.89; *p* = 0.007; Table 4, Figure 3). Elevated CA19-9 value independently predicted worse overall survival (HR 1.39, 95% CI 1.13–1.71; *p* = 0.002), indicating that normal CA19-9 (≤37 U/mL) carried independent favorable prognostic information. N-positive disease (HR 1.40, 95% CI 1.12–1.75; *p* = 0.003) and T stage (HR 1.32, 95% CI 1.14–1.53; *p* < 0.001) also remained independent adverse prognostic factors. Female sex was not independently significant (HR 0.85, 95% CI 0.71–1.01; *p* = 0.071).

In Table 5 and Table 6, the sensitivity model showed that adding adjuvant therapy (*N* = 588; deaths = 464), age < 55 years remained independently favorable (HR 0.70, 95% CI 0.52–0.95; *p* = 0.022), elevated CA19-9 remained independently adverse (HR 1.50, 95% CI 1.22–1.86; *p* < 0.001), and adjuvant therapy was independently associated with improved overall survival (HR 0.42, 95% CI 0.34–0.52; *p* < 0.001). Sensitivity model results are provided in Tables S1 and S2 (Supplementary Materials).

## 4. Discussion

This multicenter cohort addresses a common clinical question in patients undergoing surgery for PDAC: should younger age be considered a warning sign for worse prognosis? The data indicate the opposite. Younger age was independently associated with better overall survival, while non-elevated CA19-9 value carried independent favorable prognostic information. The primary model retained baseline and pathological variables that clinicians already use at the bedside, improving interpretability.

This result should not be interpreted as evidence that early-onset PDAC is biologically equivalent or prognostically more favourable to conventional-onset disease. Molecular and clinical studies have described features that may distinguish tumors arising in younger patients [17,18,19,20]. In the present pooled dataset, mutation data for major PDAC driver genes such as KRAS, TP53, SMAD4, and CDKN2A were not available in a harmonized manner, so age-related molecular comparisons could not be performed. However, biological distinctiveness and postoperative prognosis are not equivalent. In a surgically selected cohort, survival reflects the combined effects of tumor biology, anatomical stage, postoperative recovery, systemic therapy, and competing mortality. A younger patient may have more aggressive tumor biology but still have better treatment tolerance and fewer competing risks.

Our data are consistent with the emerging surgical literature on early-onset PDAC. Leonhardt et al. reported that resected early-onset pancreatic cancer frequently presented with advanced disease, but survival was primarily governed by AJCC stage and resection margin status [8]. Mughal et al. similarly showed that young-onset disease is a relevant subgroup but cannot be reduced to chronological age alone [9]. Our analysis extends this concept by showing that, after accounting for harmonized pathological and treatment variables, the age effect is not adverse.

Other studies may help explain why age-related associations differ across studies. Tschaidse et al., using the German StuDoQ|Pancreas registry, showed that older age was associated with higher major morbidity, mortality, and failure-to-rescue after pancreatic surgery, whereas pancreas-specific complications did not uniformly increase with age [10]. Matsui et al. also reported poorer survival across older age categories after radical resection, but such analyses inevitably blend cancer-related risk, perioperative resilience, and postoperative treatment delivery [11].

CA19-9 status was clinically informative. Patients with normal CA19-9 value had longer survival, and elevated CA19-9 remained independently associated with worse outcome. The combination of younger age and not elevated CA19-9 value identifies a more favorable prognostic profile. CA19-9 is not a perfect biomarker—it may be influenced by biliary obstruction, inflammation, Lewis antigen status, and timing of measurement—but when available at baseline, it provides prognostic information that age alone cannot capture.

Adjuvant therapy showed a strong favorable association with overall survival in the sensitivity analysis. Receipt of adjuvant therapy is influenced by postoperative complications, performance status, nutritional recovery, and early relapse; the effect should therefore be read as a marker of delivered multimodality care. Part of the apparent survival advantage in younger patients may be mediated through higher rates of adjuvant therapy completion, which is why including it in the sensitivity model is informative but may also partially adjust away a causal mechanism.

Regarding the age threshold, the most widely adopted cutoff in large database studies and recent meta-analyses of early-onset PDAC is 50 years [4,5,7]. Our 55-year definition is supported by translational evidence—Tsang et al. found that the molecular landscape of PDAC changes meaningfully across age, with a boundary closer to 55 years [12]—but it encompasses patients aged 50–54 years who would be classified as conventional-onset under the 50-year definition. In a sensitivity analysis using a <50-year threshold, 38 patients in the pooled analytic cohort were aged < 50 years; the direction of effect remained non-adverse (Table S3).

Recent work on long-term survival and treatment personalization after PDAC resection further supports a multidimensional view of prognosis [15,16,21]. Javed et al. showed that the impact of established prognostic factors after resection is time-varying [22], suggesting that baseline variables may not exert constant effects throughout follow-up. Verkolf et al. demonstrated that true 10-year survival remains uncommon even after resection and pathological re-examination [23].

Several limitations must be acknowledged. First, the retrospective design and multicenter data pooling introduce residual confounding that cannot be fully eliminated despite harmonization across participating centers. Second, resection margin status (R0/R1) was not sufficiently standardized across centers for robust inclusion in the multivariable model; margin status is a recognized major prognostic factor in resected PDAC, and its absence may confound the observed associations, particularly if younger patients disproportionately underwent surgery at high-volume centers with differing margin assessment practices. Third, the age threshold of <55 years differs from the 50-year cutoff most widely adopted in large database studies and meta-analyses [4,5,7]; patients aged 50–54 years would be classified as conventional-onset under that definition. A sensitivity analysis using a <50-year threshold included 38 patients aged < 50 years in the pooled analytic cohort (18 from DB1 and 20 from DB2). The direction of effect remained non-adverse, but the association was not statistically significant after adjustment for sex, N-positive disease, T stage, and CA19-9 > 37 U/mL (HR 0.86, 95% CI 0.60–1.25; *p* = 0.440; Table S3), likely reflecting the small size of the <50-year subgroup. Fourth, CA19-9 was missing in 57 patients (8.2%) and may not have been measured at a strictly standardized perioperative timepoint across all centers. Furthermore, Lewis antigen non-secretor status, present in approximately 5–10% of the general population and associated with permanently undetectable CA19-9 independent of tumor burden, was not systematically available and may have caused non-differential misclassification of some patients as having normal CA19-9, which would bias the CA19-9 hazard ratio toward the null. Notably, median CA19-9 did not differ significantly between age groups in the present cohort (118.7 U/mL in patients aged < 55 years versus 157.0 U/mL in those aged ≥ 55 years; *p* = 0.411), and the proportion with elevated CA19-9 was similarly distributed (67.6% versus 70.9%; *p* = 0.575). Thus, the observed age-survival association is unlikely to be explained by a systematic difference in CA19-9 distribution between age groups. Fifth, the young-age subgroup comprised only 70 patients with 50 events, limiting statistical power and yielding an imprecise point estimate (HR 0.66, 95% CI 0.49–0.89); prospective validation in larger, dedicated early-onset cohorts is warranted. Sixth, adjuvant chemotherapy regimen was not available for harmonized analysis; the strong favorable association of adjuvant therapy with overall survival (HR 0.42 in sensitivity analysis) should be interpreted as reflecting delivered multimodality care rather than a regimen-specific effect, given the differing magnitudes of benefit reported for gemcitabine-based versus FOLFIRINOX-based adjuvant regimens in contemporary randomized trials. Seventh, data on disease recurrence and disease-free survival were not consistently available across participating centers; overall survival was therefore used as the sole endpoint, which does not distinguish between early relapse and non-cancer mortality. Eighth, a formal test of interaction between age group and CA19-9 status was not performed; whether the favorable effect of younger age is modified by CA19-9 level remains to be established. Finally, center-level heterogeneity could not be robustly adjusted for in multivariable models given the sample sizes per participating institution; a multilevel or frailty model approach should be considered in future analyses with larger cohorts. With regard to missing covariate data, 22 of 696 patients (3.2%) were excluded from multivariable models; given this low proportion and the absence of an obvious systematic pattern, complete-case analysis was considered appropriate and formal multiple imputation was not performed.

## 5. Conclusions

Among surgically managed PDAC patients, young age (<55 years) and not elevated CA19-9 value carried independent favorable prognostic information in the primary multivariable Cox model. N-positive disease, higher T stage, and elevated CA19-9 identified patients with worse outcomes. Sensitivity analysis confirmed that age and CA19-9 associations persisted after adding adjuvant therapy. Age-based prognostic interpretation in resected PDAC should be contextualized by tumor burden, CA19-9 status, and postoperative treatment delivery. Future prospective studies with molecular annotation are needed to determine whether early-onset PDAC constitutes a biologically and clinically distinct entity that warrants individualized management.

## Figures and Tables

**Table 1:** Patient and tumor characteristics by age group.

Characteristic	Age < 55	Age ≥ 55	*p*-Value
Patients, *n*	70	626	
Female sex	25 (35.7%)	314 (50.2%)	0.022
CA19-9, U/mL	118.7 (32.2–373.0)	157.0 (28.4–681.0)	0.411
CA19-9 > 37 U/mL	46 (67.6%)	405 (70.9%)	0.575
N-positive disease	53 (76.8%)	465 (76.6%)	0.970
T stage	3.0 (2.0–3.0)	3.0 (2.0–3.0)	0.469
Adjuvant therapy	54 (80.6%)	397 (72.4%)	0.154

IQR: interquartile range; OS: overall survival.

**Table 2:** Overall survival according to CA19-9 status.

Marker	Group High/Positive	Group Low/Negative	*N*	Median OS High/Positive	Median OS Low/Negative	12-Month OS High/Positive	12-Month OS Low/Negative	24-Month OS High/Positive	24-Month OS Low/Negative	Log-Rank *p*
CA19-9 > 37 U/mL	>37 (*n* = 451)	≤37 (*n* = 188)	639	19.0	31.5	64.0	82.2	42.5	60.5	<0.001

OS: overall survival.

**Table 3:** Univariable Cox proportional hazards models.

Variable	*N*	Deaths	HR	95% CI	*p*-Value
Age < 55 years	696	524	0.71	0.53–0.95	0.022
Female sex	695	524	0.87	0.73–1.04	0.120
N-positive disease	676	517	1.59	1.29–1.97	<0.001
T stage	679	519	1.27	1.17–1.39	<0.001
CA19-9 > 37 U/mL	639	492	1.55	1.27–1.90	<0.001
Adjuvant therapy yes	615	477	0.53	0.43–0.65	<0.001

HR: hazard ratio; CI: confidence interval.

**Table 4:** Primary multivariable Cox model (age, sex, N status, T stage, CA19-9).

Variable	*N*	Deaths	HR	95% CI	*p*-Value
Age < 55 years	621	486	0.66	0.49–0.89	0.007
Female sex	621	486	0.85	0.71–1.01	0.071
N-positive disease	621	486	1.40	1.12–1.75	0.003
T stage	621	486	1.32	1.14–1.53	<0.001
CA19-9 > 37 U/mL	621	486	1.39	1.13–1.71	0.002

HR: hazard ratio; CI: confidence interval.

**Table 5:** Sensitivity multivariable Cox model including adjuvant therapy.

Variable	*N*	Deaths	HR	95% CI	*p*-Value
Age < 55 years	588	464	0.70	0.52–0.95	0.022
Female sex	588	464	0.83	0.69–1.00	0.051
N-positive disease	588	464	1.42	1.13–1.78	0.003
T stage	588	464	1.42	1.23–1.65	<0.001
CA19-9 > 37 U/mL	588	464	1.50	1.22–1.86	<0.001
Adjuvant therapy yes	588	464	0.42	0.34–0.52	<0.001

HR: hazard ratio; CI: confidence interval.

**Table 6:** Overall survival according to receipt of adjuvant therapy.

Group	N	Deaths	Median OS Months	12-Month OS %	24-Month OS %	Log-Rank *p*
Adjuvant yes	451	337	27.0	79.5	54.8	<0.001
Adjuvant no	164	140	11.6	46.3	30.7	<0.001

OS: overall survival.

## Data Availability

The data are not publicly available due to privacy and ethical restrictions involving clinical patient-level data.

## Supplementary Materials

The following supporting information can be downloaded at https://www.mdpi.com/article/10.3390/life16091499/s1, Table S1. Sensitivity multivariable Cox model including adjuvant therapy (*N* = 588). Table S2. Overall survival according to receipt of adjuvant therapy. Table S3. Sensitivity multivariable Cox model using a <50-year age threshold. Table S4. Exploratory overall survival analysis across four age categories.
