# Supplementary material for: Young Age Is Associated with Improved Survival After Resection of Non-Metastatic Pancreatic Ductal Adenocarcinoma
Source: Life (Basel). 2026 Sep 8;16(9):1499. doi: 10.3390/life16091499 (PMC13608593; doi:10.3390/life16091499)
Supplement: Supplementary file 1 [file life-16-01499-s001.zip › life-4521333-supplementary.pdf]

Supplementary Materials

Table S1. Sensitivity multivariable Cox model including adjuvant therapy (N=588).

Model	Variable	N	Deaths	HR	95% CI	p-value
Sensitivity: primary + adjuvant therapy	Age <55 years	588	464	0.70	0.52-0.95	0.022
Sensitivity: primary + adjuvant therapy	Female sex	588	464	0.83	0.69-1.00	0.051
Sensitivity: primary + adjuvant therapy	N-positive disease	588	464	1.42	1.13-1.78	0.003
Sensitivity: primary + adjuvant therapy	T stage	588	464	1.42	1.23-1.65	<0.001
Sensitivity: primary + adjuvant therapy	CA19-9 >37 U/mL	588	464	1.50	1.22-1.86	<0.001
Sensitivity: primary + adjuvant therapy	Adjuvant therapy yes	588	464	0.42	0.34-0.52	<0.001

Table S2. Overall survival according to receipt of adjuvant therapy.

Group	N	Deaths	Median OS months	12-month OS %	24-month OS %	log-rank p
Adjuvant yes	451	337	27.0	79.5	54.8	<0.001
Adjuvant no	164	140	11.6	46.3	30.7	<0.001

Table S3. Sensitivity multivariable Cox model using a <50-year age threshold.

Model	Variable	N	Deaths	HR	95% CI	p-value
Sensitivity: age <50 threshold; primary	Age <50 years	621	486	0.86	0.60-1.25	0.440

model + CA19-9 >37						

Note: The <50-year sensitivity model is reported as an exploratory analysis. Only 38 patients in the pooled analytic cohort were aged <50 years

Table S4. Exploratory overall survival analysis across four age categories.

Age group	N	Deaths	Median OS months	12-month OS %	24-month OS %	Adjusted HR vs <55	95% CI	p-value
<55	70	50	34.4	83.8	65.3	Reference		
55-64	157	113	25.3	73.8	52.7	1.41	1.01-1.99	0.046
65-74	280	206	24.0	70.6	49.3	1.31	0.96-1.80	0.092
>=75	189	155	17.1	60.6	36.8	2.12	1.52-2.96	<0.001

Note: Adjusted HRs are from a Cox model including age category, sex, N-positive disease, T stage, and CA19-9 >37 U/mL. The <55-year group was used as the reference category; overall age-category log-rank p<0.001.
